# Mice Rescued from Severe Malaria Are Protected against Renal Injury during a Second Kidney Insult

**DOI:** 10.1371/journal.pone.0093634

**Published:** 2014-04-15

**Authors:** Thiago P. Abreu, Leandro S. Silva, Christina M. Takiya, Mariana C. Souza, Maria G. Henriques, Ana Acacia S. Pinheiro, Celso Caruso-Neves

**Affiliations:** 1 Instituto de Biofísica Carlos Chagas Filho, Universidade Federal do Rio de Janeiro, Rio de Janeiro, RJ, Brazil; 2 Instituto de Tecnologia em Fármacos, Fundação Oswaldo Cruz, Rio de Janeiro, RJ, Brazil; 3 Instituto Nacional para Pesquisa Translacional em Saúde e Ambiente na Região Amazônica, Conselho Nacional de Desenvolvimento Científico e Tecnológico/MCT, Rio de Janeiro, RJ, Brazil; 4 Instituto Nacional de Ciência e Tecnologia em Biologia e Bioimagem, Conselho Nacional de Desenvolvimento Científico e Tecnológico/MCT, Rio de Janeiro, RJ, Brazil; The University of Manchester, United Kingdom

## Abstract

Malaria is a worldwide disease that leads to 1 million deaths per year. *Plasmodium falciparum* is the species responsible for the most severe form of malaria leading to different complications. Beyond the development of cerebral malaria, impairment of renal function is a mortality indicator in infected patients. Treatment with antimalarial drugs can increase survival, however the long-term effects of malaria on renal disease, even after treatment with antimalarials, are unknown. The aim of this study was to evaluate the effect of antimalarial drug treatment on renal function in a murine model of severe malaria and then evaluate kidney susceptibility to a second renal insult. Initially, mice infected with *Plasmodium berghei* ANKA achieved 20% parasitemia on day 5 post infection, which was completely abolished after treatment with 25 mg/kg artesunate and 40 mg/kg mefloquine. The treatment also decreased plasma creatinine levels by 43% and partially reversed the reduction in the glomerular filtration rate induced by infection. The urinary protein/creatinine ratio, collagen deposition, and size of the interstitial space decreased by 75%, 40%, and 20%, respectively, with drugs compared with untreated infected animals. In infected-treated mice that underwent a second renal insult, the plasma creatinine level decreased by 60% and the glomerular filtration rate increased compared with infected animals treated only with antimalarials. The number of glomerular cells, collagen deposition and the size of the interstitial space decreased by 20%, 39.4%, and 41.3%, respectively, in the infected group that underwent a second renal insult compared with the infected-treated groups. These functional and structural data show that renal injury observed in a murine model of severe malaria is partially reversed after antimalarial drug treatment, making the kidney less susceptible to a second renal insult.

## Introduction

Malaria is a global disease that affects about 500 million people every year; about 1.5 million people die from malaria every year [1]. The life cycle of the parasite involves hepatic and erythrocytic stages; the clinical manifestations follow erythrocyte rupture [2]. Among the five species of parasites that can infect humans, *Plasmodium falciparum* is the most severe form leading to the development of cerebral damage (cerebral malaria), respiratory distress, and kidney injury [3].

The development of experimental cerebral malaria (ECM) coincides with the accumulation of the parasite in different organs such as the heart, lungs, spleen, liver, gastrointestinal tract, and the kidneys [4]. A high level of plasma creatinine is one of the major factors associated with mortality in humans infected with *Plasmodium falciparum*, indicating that impairment of renal function is a great risk factor for these patients infected with malaria [5]. It has been proposed that renal injury depends on the cytoadherence of infected erythrocytes to the microvasculature and the host immune response, which play a role in tubular and glomerular damage, respectively [3].

Usually, survival of mice infected with *Plasmodium berghei* ANKA (PbA), a murine model of severe malaria, is associated with a decrease in cerebral edema after treatment with antimalarial drugs [6]. Soniram et al [7] showed that treatment of PbA-infected albino mice concomitantly with artesunate (100 mg/kg/day) and chloroquine (100 mg/kg/day) attenuates renal interstitial infiltration of mononuclear cells and tubular necrosis [7]. These authors measured kidney injury immediately after treatment. However, it has not yet been determined whether the recovery from kidney injury is maintained even after the end of treatment. Although antimalarial drugs show beneficial effects on survival, the long-term effect on renal function remains unclear.

Another important factor is the tubule interstitial injury, which is a crucial step in the progression of renal disease. In this context, tubule interstitial lesions are observed in acute kidney injury induced by malaria infection [7]. Malaria and septic shock presents similarities mainly in relation to a long-term immunosuppressive state [8]. Recently, our group showed that sepsis-surviving mice are more susceptible to a secondary kidney insult [9]. On the other hand, little is known about kidney susceptibility to a second insult in patients who have survived malaria or in a malaria animal model. In the present study, we aimed to determine the correlation between malaria and acute kidney injury (AKI), using PbA-infected mice, a well-known murine model of severe malaria.

Two main questions are addressed: (1) Does the treatment of PbA-infected mice with antimalarial drugs lead to complete recovery from AKI even after treatment has been stopped? (2) Are PbA-infected mice treated with antimalarial drugs more susceptible to a second kidney insult? The BSA challenge tubule interstitial injury animal model is used as a second kidney insult. In this animal model, the glomerular flow rate (GFR) is not changed.

## Materials and Methods

### Ethics Statement

This work was carried out in strict accordance with the recommendations in the Guide for the Care and Use of Laboratory Animals of the National Institutes of Health. The protocol was approved by the Institutional Ethics Committee of Federal University of Rio de Janeiro (permit number CEUA-CCS-098) and by The Committee on Ethical Use of Laboratory Animals of Fundação Oswaldo Cruz (permit number L004/08).

### Animals

C57BL/6 male mice (6–8 weeks old) were used. When indicated, the animals were (a) infected or not by intraperitoneal injection of 10^6^ erythrocytes infected with PbA in a final volume of 200 µl, as a model of severe malaria; and/or (b) animals were subjected to intraperitoneal injections of saline or 10 g/kg/day bovine serum albumin (BSA) for 7 days, a BSA challenge tubule interstitial injury animal model [10]. All animals were allocated to metabolic cages and kept in an air-conditioned environment (22–24°C) with 12-h alternating periods of light/dark and free access to food and fresh water.

### Experimental Protocols

**Figure 1:**
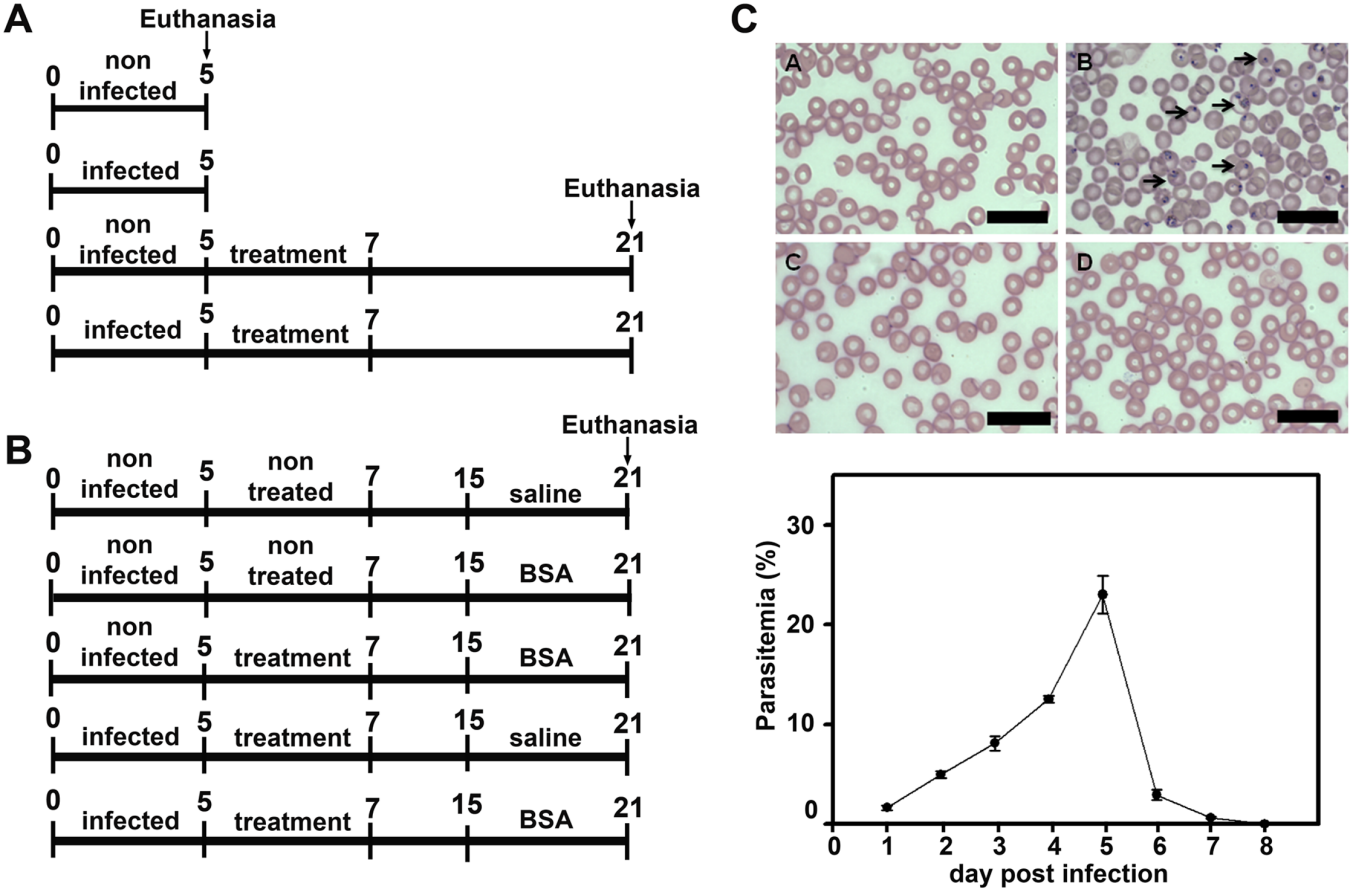
Experimental design. (**A**) C57BL/6 mice were separated into four experimental groups that were euthanized on day 5 (5d) or day 21 (21d) post infection (p.i.): noninfected group (control_5d_); mice infected with 10^6^ infected red blood cells euthanized on day 5 p.i. (infected_5d_); noninfected mice that received treatment with antimalarials (control+treated_21d_); and the *Plasmodium berghei* ANKA (PbA)-infected group treated with antimalarials and euthanized on day 21 p.i. (infected+treated_21d_). (**B**) Additional experimental groups that were euthanized on day 21 underwent or not a second kidney insult induced with intraperitoneal injections of bovine serum albumin (BSA): noninfected group (control_21d_); noninfected mice with BSA-induced renal injury (control+BSA_21d_); noninfected mice that received treatment with antimalarials and subsequent BSA injections (control+treated+BSA_21d_); PbA-infected mice treated with antimalarials (infected+treated_21d_); and PbA-infected mice that were treated and subsequently underwent a second kidney insult (infected+treated+BSA_21d_). (**C**) Parasitemia was determined from blood smears stained with Diff-Quick in all infected groups on day 1 (**A**), day 5 (**B**), day 8 (**C**), and day 21 (**D**) p.i.

**Figure 2:**
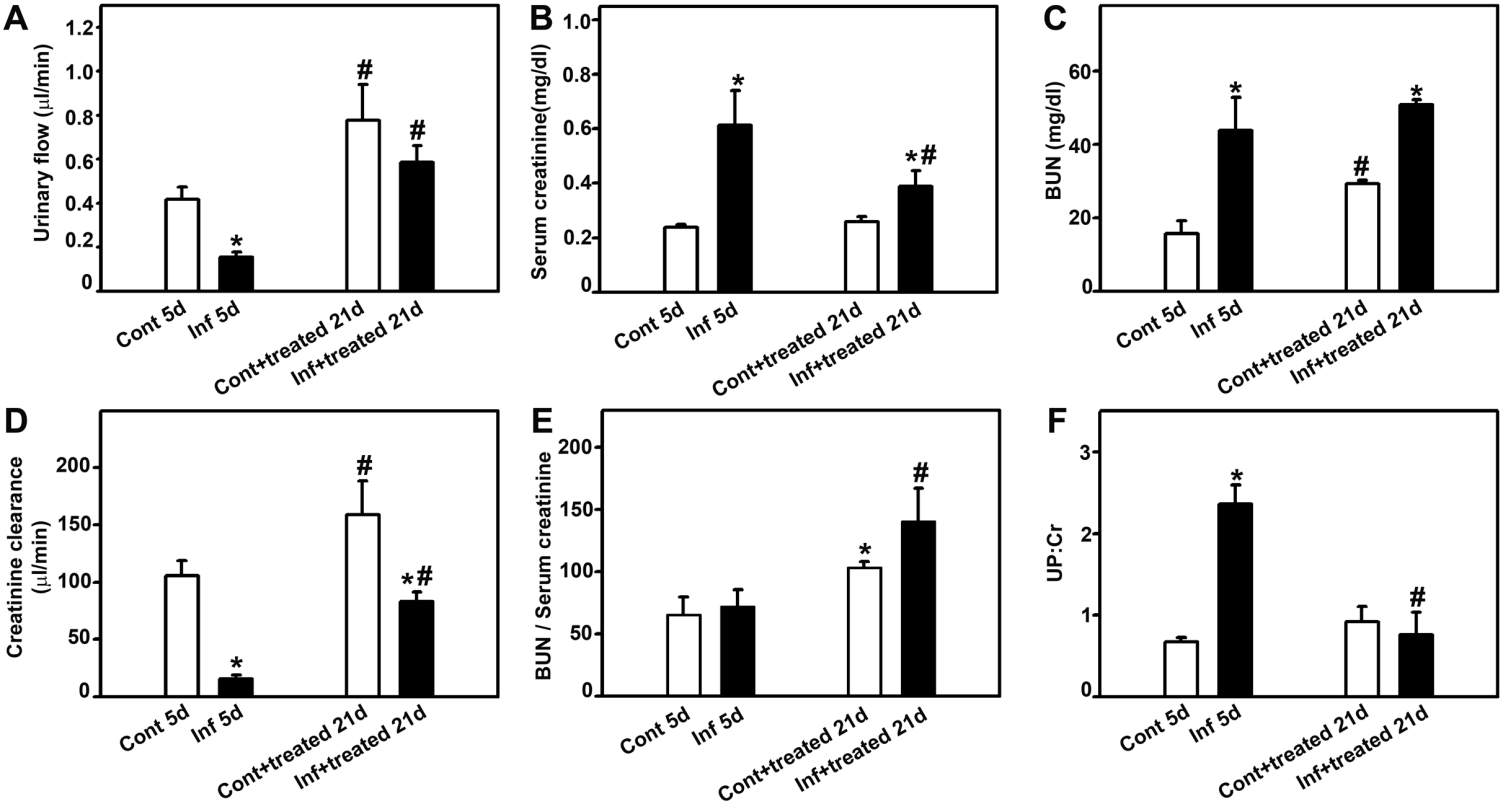
Effect of antimalarial treatment on malaria-induced renal damage. Animals were euthanized at day 5 or 21 post infection and plasma and urine samples were collected for analysis of renal function. Analysis of (**A**) urinary flow, (**B**) serum creatinine, (**C**) blood urea nitrogen (BUN), (D) creatinine clearance (CCr), (**E**) BUN/serum creatinine ratio, and (**F**) urinary protein/creatinine (UPC) ratio in different experimental groups as depicted in the figure. *Statistically significant compared with control_5d_ or control+treated_21d_. #Statistically significant compared with control_5d_ or infected_5d_ (*P*<0.05).

Two experimental protocols were developed: (1) to determine renal function in PbA-infected mice treated or not with antimalarial drugs (Figure 1A); (2) to test if mice that have recovered from infection after treatment with antimalarial drugs are more or less susceptible to a secondary kidney insult (Figure 1A). Six animals were used for each group. In the first experimental protocol, the animals were randomly divided into four groups: (1) noninfected and untreated mice (control_5d_); (2) infected and untreated mice (infected_5d_); (3) noninfected mice that were treated with antimalarial drugs following the protocol described by Clemmer et al [6]. Animals were treated with 25 mg/kg/day artesunate on days 5, 6, and 7 post infection (p.i.) and, concomitantly with 40 mg/kg/day mefloquine on day 7 p.i. (Figure 1A and B). (control+treated_21d_); (4) infected and treated mice (infected+treated_21d_). Untreated mice and treated mice were euthanized at day 5 (at the onset of cerebral malaria symptoms) and 21, respectively.

In the second experimental protocol, the animals were randomly divided into five groups: (1) noninfected, untreated, and no BSA i.p. injection (control_21d_); (2) noninfected, untreated, and BSA i.p. injection (control+BSA_21d_); (3) noninfected, treated, and BSA i.p. injection (control+treated+BSA_21d_); (4) infected, treated, and no BSA i.p. injection mice (infected+treated_21d_); (5) infected, treated, and BSA i.p., injection (infected+treated+BSA_21d_). All animals were euthanized on day 21 p.i.

The animal condition was checked daily and the animals were humanely euthanized when they met certain clinical cerebral malaria symptoms such as paralysis, deviation of the head, ataxia, convulsions and coma. For the euthanasia procedure, animals were anesthetized with ketamine (80 mg/kg body weight) and xylazine (5 mg/kg body weight) before blood collection via cardiac puncture.

### Assessment of Blood Parasitemia

Parasitemia was determined daily in all infected groups throughout the period in the metabolic cage (Figure 1C). For this, blood smears were prepared with a drop of blood extracted from the animal’s tail and stained with Diff-Quick. The percentage of parasitemia was described as the number of parasitized red blood cells (pRBC) in 100 erythrocytes after analysis of at least five random microscopic fields. On day 5 p.i., when parasitemia reached 20%, the animals were treated with antimalarial drugs and the parasitemia was completely abolished up to day 21. Infected animals that did not receive any treatment died on day 6 p.i. (data not shown).

### Measurement of Renal Function

After an adaptation period of 24 h, urine volume was determined and collected for the last 24 h before euthanasia on days 5 and 21. Urine samples were clarified at 600×*g* for 5 min, and the supernatant was separated and stored at −20°C until use. The levels of protein, urea, and creatinine were determined colorimetrically in urine samples using specific Gold Analisa kits (498M, 427E, and 335, respectively). Blood samples were collected via cardiac puncture in heparinized tubes and centrifuged at 600×*g* for 5 min to separate plasma. In these samples, the level of plasma creatinine and blood urea nitrogen (BUN) were determined. The glomerular filtration rate (GFR) was determined by creatinine clearance (CCr).

### Kidney Histology

On the days of euthanasia, the kidneys were analyzed histologically as described by Landgraf et al [11]. Briefly, after perfusion with saline and 4% paraformaldehyde, kidneys were sectioned midfrontally into two pieces and immersed in Gendre fixative solution. The sections were fixed in 10% buffered formalin for 48 h and subsequently embedded in paraffin; 4-µm-thick sections were prepared for analysis of the glomerular cell number and interstitial space using periodic acid−Schiff staining. In addition, 7-µm-thick sections were cut to measure the deposition of collagen fibers with Picrosirius Red staining. The percentage of collagen deposition was obtained by dividing the area measured by the total area of the tissue. All analyses were performed with Image-Pro Plus on 20 photomicrographs in a light microscope (Eclipse E800, Nikon). To avoid individual interference in the results, the samples were numbered and the kidney histology analysis was conducted blind.

### Statistical Analysis

The results are expressed as means ± standard error. Differences between the control and experimental groups were analyzed by two-way analysis of variance followed by the Newman-Keuls test for multiple comparisons.

## Results

### Correlation between Malaria and Renal Failure in PbA-infected Mice

Initially, possible correlation between malaria and renal injury was tested (Figure 2). The urinary flow decreased (Figure 2A) and serum creatinine (Figure 2B) and BUN (Figure 2C) increased in the infected_5d_ group. Urine creatinine was unchanged in all groups tested (data not shown). These effects are correlated to the decrease in CCr, a marker of the GFR (Figure 2D). The treatment of infected mice with antimalarial drugs (infected+treated_21d_ group) reversed all these effects. However, the antimalarial treatment did not reverse the effect of infection on the BUN level. In addition, antimalarial treatment (control+treated_21d_) per se increased the urinary flow, BUN, and CCr, but the serum creatinine level was not changed. The ratio between BUN and serum creatinine was increased in both the control+treated_21d_ and infected+treated_21d_ groups (Figure 2E) indicating that the treatment with antimalarial drugs could affect urea production in the liver.

The urinary protein/creatinine (UPC) ratio is a well-established marker of renal injury [12]. We observed that the value of the UPC ratio was three times higher in the infected_5d_ group than in the control_5d_ group (Figure 2F). This increase was abolished when the animals were treated with antimalarial drugs (infected+treated group). It is relevant that treatment of noninfected animals (control+treated_21d_ group) did not change the UPC ratio, indicating that antimalarial treatment does not promote kidney injury. These results were obtained by analyzing the profile of renal protein excretion using sodium dodecyl sulfate polyacrylamide gel electrophoresis (Figure S1).

**Figure 3:**
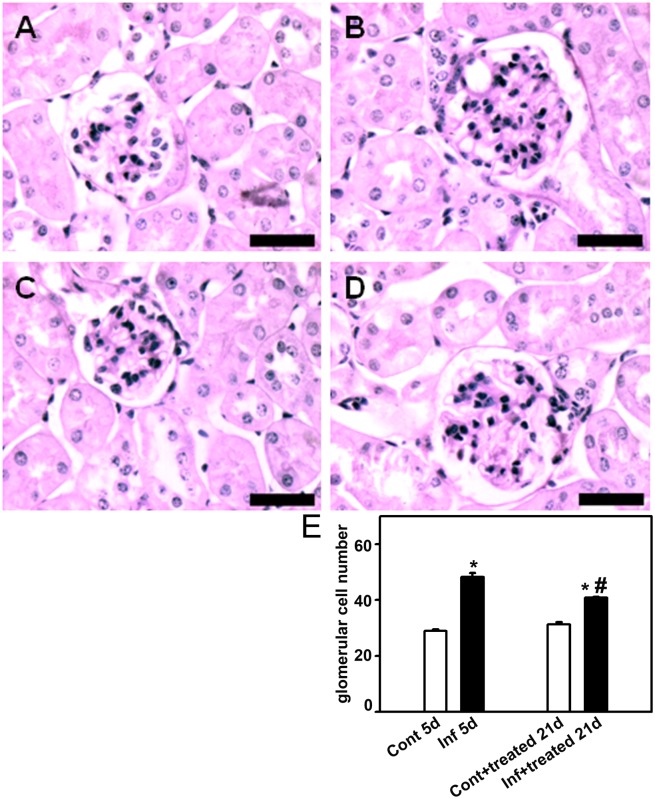
Effect of antimalarial treatment on the number of glomerular cells. Animals were euthanized on day 5 or day 21 p.i., perfused, and the kidneys were collected for histologic analysis. The number of glomerular cells in the renal cortex was visualized using periodic acid−Schiff. Representative photomicrography of noninfected mice (control_5d_) (**A**), 5 days p.i. (infected_5d_) (**B**), noninfected mice that received treatment with antimalarial drugs (control+treated_21d_) (**C**), and infected mice that received treatment with antimalarial drugs (infected+treated_21d_) (**D**) (*n* = 6 per group). Bar = 40 µm. The number of glomerular cells is quantified in (*E*). The results are expressed as means ± standard error. *Statistically significant compared with control_5d_ or control+treated_21d_. #Statistically significant compared with control_5d_ or infected_5d_ (*P*<0.05).

**Figure 4:**
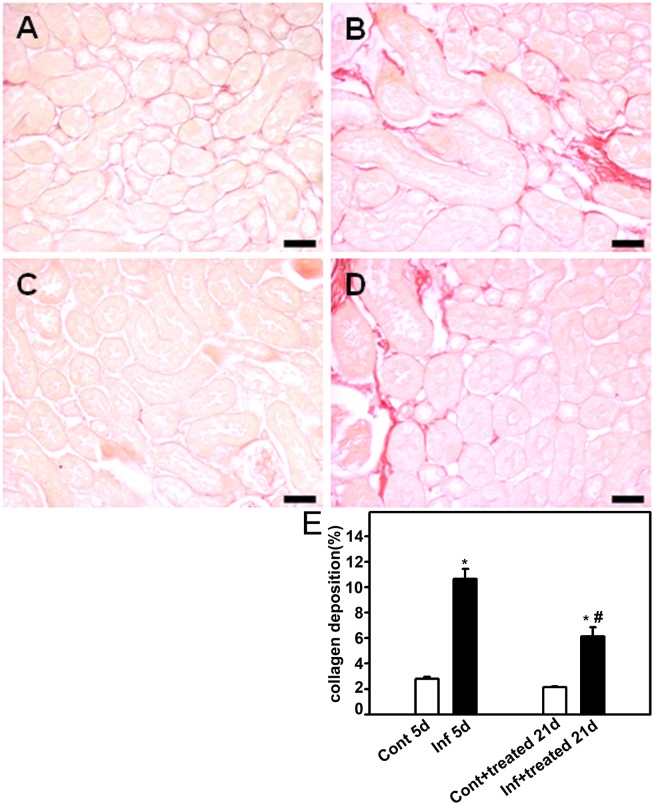
Effect of antimalarial treatment on collagen deposition in the renal cortex of mice. Animals were euthanized on day 5 or 21 post infection (p.i.), perfused, and the kidneys were collected for histologic analysis. Collagen deposition in the renal cortex was visualized using Picrosirius Red stain. Representative photomicrography of noninfected mice (control_5d_) (**A**), 5 days p.i. (infected_5d_) (**B**), noninfected mice that received treatment with antimalarial drugs (control+treated_21d_) (**C**), and infected mice that received treatment with antimalarials (infected+treated_21d_) (**D**) (*n* = 6 per group). Bar = 20 µm. Collagen deposition is quantified in (**E**). Values are expressed as a percentage of collagen deposition per area (means ± standard error). *Statistically significant compared with control_5d_ or control+treated_21d_. #Statistically significant compared with control_5d_ or infected_5d_ (*P*<0.05).

**Figure 5:**
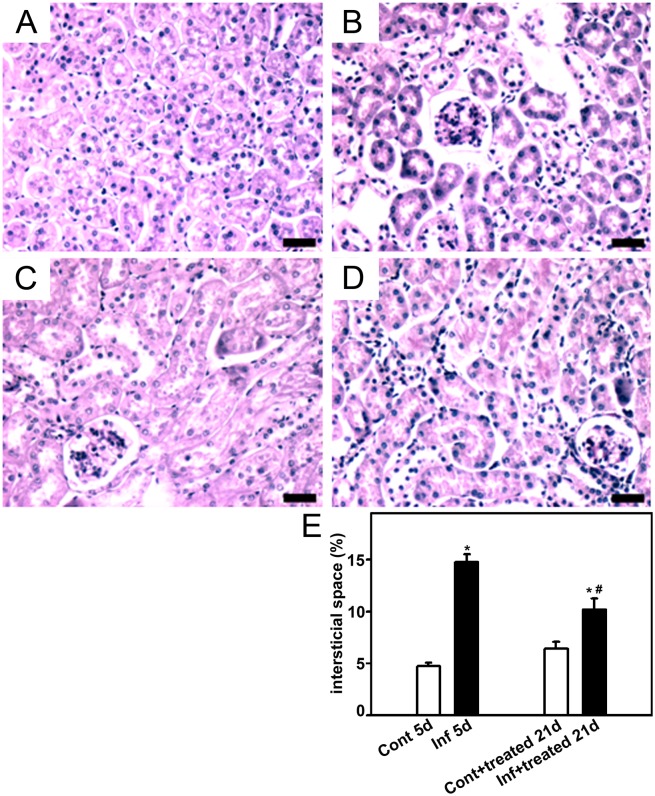
Effect of antimalarial treatment on the size of the interstitial space. Animals were euthanized on day 5 or 21 post infection (p.i.), perfused, and the kidneys were collected for histological analysis. The size of the interstitial space in the renal cortex was visualized using periodic acid−Schiff. Representative photomicrography of noninfected mice (control_5d_) (**A**), 5 days p.i. (infected_5d_) (**B**), noninfected mice that received treatment with antimalarial drugs (control+treated_21d_) (**C**), and infected mice that received treatment with antimalarial drugs (infected+treated_21d_) (**D**) (*n* = 6 per group). Bar = 20 µm. The size of the interstitial space is quantified in (**E**). Values are expressed as a percentage of interstitial space per area (means ± standard error). *Statistically significant compared with control_5d_ or control+treated_21d_. #Statistically significant compared with control_5d_ or infected_5d_ (*P*<0.05).

To establish a correlation between the improvement in renal function with histomorphological changes, the kidney was removed to measure the following histologic parameters: glomerular cell number, cortical collagen deposition, and cortical interstitial space. On day 5, the infected group (infected_5d_ group) showed a higher number of glomerular cells (Figure 3), increased collagen deposition (Figure 4), and more interstitial space (Figure 5). All these changes were partially reversed when mice were treated with antimalarial drugs (infected+treated_21d_). Antimalarial treatment (treated_21d_ group) did not change the renal histomorphological parameters measured (Figures 3−5). This observation reinforces the idea that the treatment does not lead to renal injury in the animal model tested.

These data show that there is a strict correlation between renal injury and malaria and that the treatment of infected mice with antimalarial drugs partially abolishes the renal injury.

### PbA-infected Mice Treated with Antimalarial Drugs are Less Susceptible to a Second Kidney Insult

To our knowledge, there are no studies on kidney susceptibility in patients who have survived malaria and are then subjected to a second kidney insult. We tested whether PbA-infected animals treated with antimalarial drugs become more or less susceptible to a second kidney insult. To evaluate this question, infected mice treated with antimalarials, received an intraperitoneal injection of BSA as described in the Materials and Methods section (Figure 1B).

**Figure 6:**
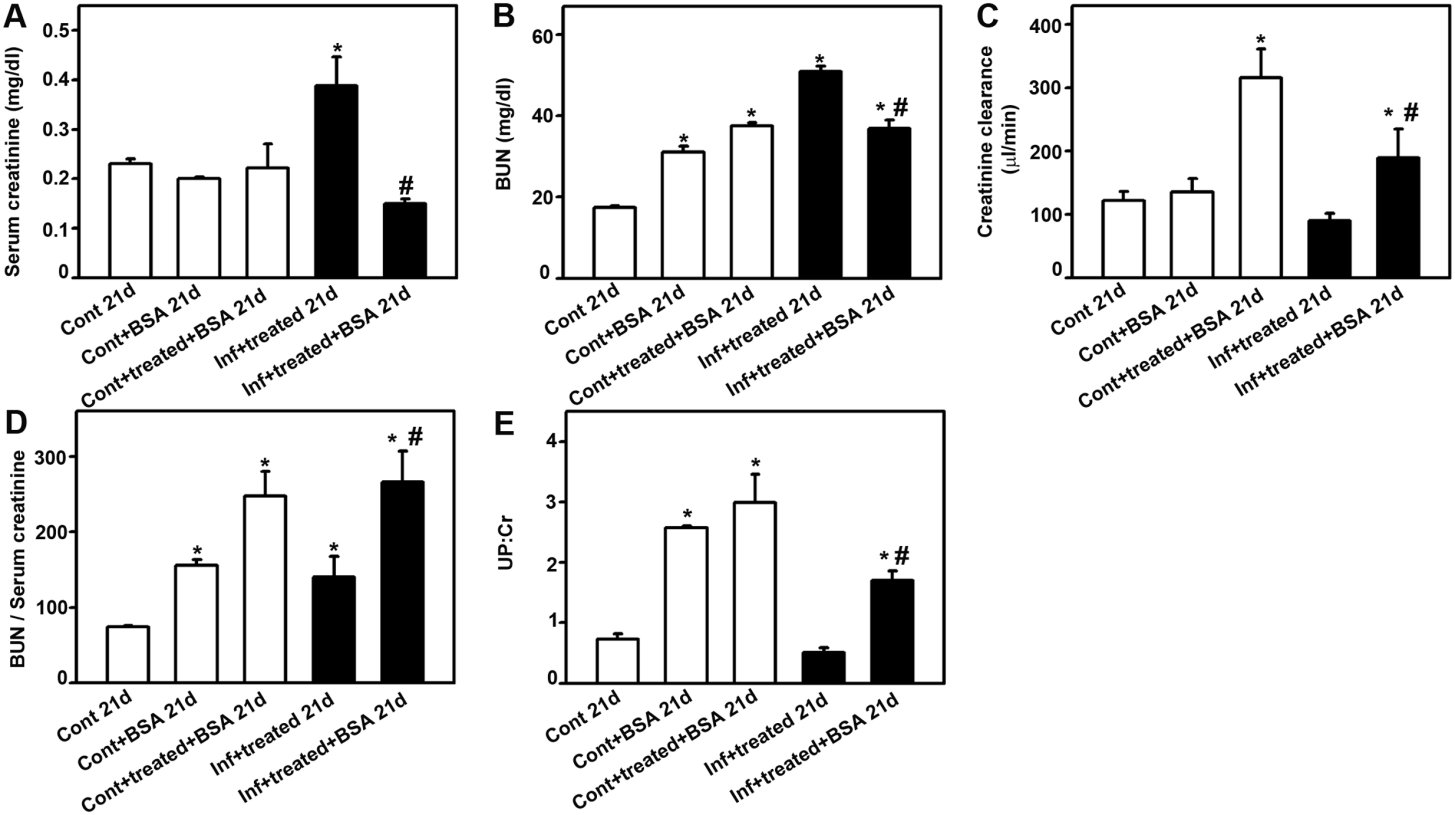
Malaria-surviving mice show less susceptibility to a second kidney insult. Animals were euthanized on day 21 post infection (p.i.) and plasma and urine samples were collected for analysis of renal function. Analysis of control_21d_, control+BSA _21d_, control+treated+BSA_21d_, infected+treated_21d_, and infected+treated+BSA_21d_: (**A**) serum creatinine, (**B**) blood urea nitrogen (BUN); (**C**) BUN/serum creatinine ratio; (**D**) creatinine clearance (CCr); and (E) urinary protein/urinary creatinine (UPC) ratio. *Statistically significant compared with control_21d_. #Statistically significant compared with infected+treated _21d_ (*P*<0.05).

Serum creatinine and BUN levels increased in PbA-infected and treated mice (infected+treated_21d_ group; Figure 6A and B) but urinary creatinine levels did not change in all groups tested (data not shown). When the infected and treated mice were subjected to a second kidney insult (infected+treated+BSA_21d_ group), the serum creatinine and BUN levels returned to the levels observed in mice subjected only to kidney insult (control+BSA_21d_ and control+treated+BSA_21d_ groups). The serum creatinine level was not changed but BUN was increased in the control+BSA_21d_ group. Moreover, the CCr value was not changed when the animals were subjected to kidney injury (control+BSA_21d_ group) but it was increased when the mice were treated with antimalarial drugs (control+treated+BSA_21d_ group; Figure 6C). The ratio of BUN to serum creatinine was increased in all groups tested, and was higher in the control+treated+BSA_21d_ and infected+treated+BSA_21d_ groups, which could indicate a liver injury as well renal damage (Figure 6D).

The UPC ratio increased in the control+BSA_21d_ and control+treated+BSA_21d_ groups compared with the control_21d_ group (Figure 6E). The UPC ratio also increased in the infected+treated+BSA_21d_ group compared with the control group. The UPC ratio was significantly lower compared with the control+treated+BSA_21d_ group. The treatment with antimalarial drugs did not change the UPC ratio per se (Figure 2E). These data indicate protection from renal injury in previously infected animals.

**Figure 7:**
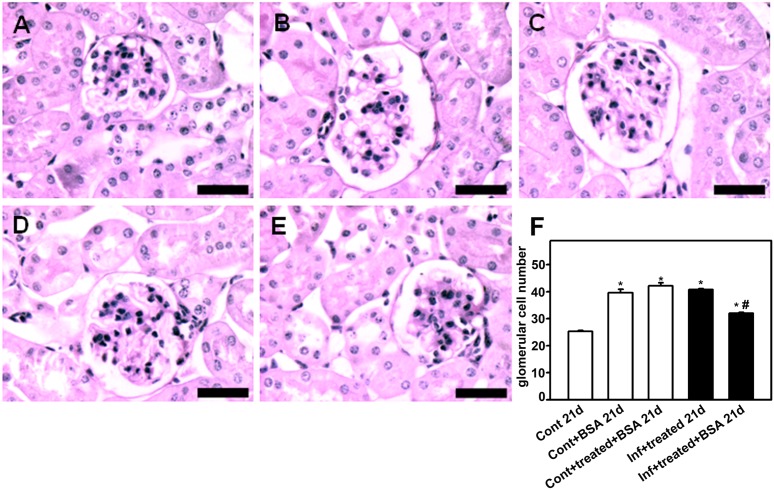
Glomerular cell number decreases in mice subjected to a second kidney insult. Animals were euthanized on day 21 post infection (p.i.), perfused, and the kidneys were collected for histologic analysis. The number of glomerular cells in the renal cortex was visualized using periodic acid−Schiff. Representative photomicrographs of (**A**) control_21d_, (**B**) control+BSA _21d_, (**C**) control+treated+BSA _21d_, (**D**) infected+treated _21d_, and (**E**) infected+treated+BSA _21d_ (*n* = 6 per group). Bar = 40 µm. The number of glomerular cells is quantified in (**F**). The results are expressed as means ± standard error. *Statistically significant compared with control_21d_. #Statistically significant compared with infected+treated _21d_ (*P*<0.05).

**Figure 8:**
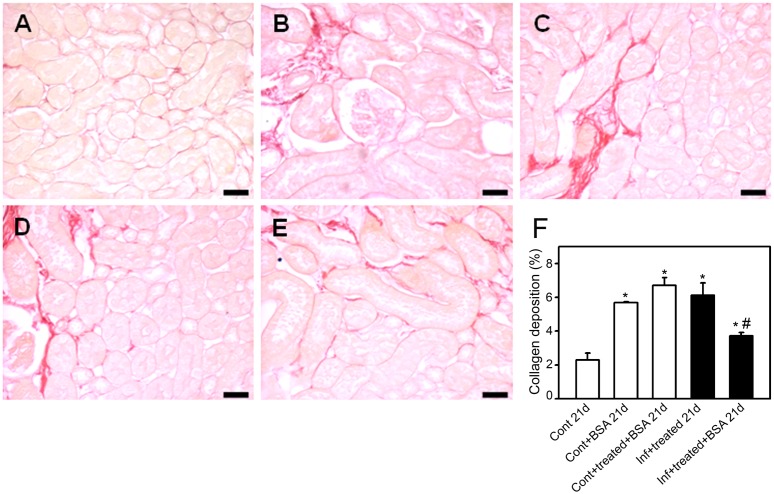
Renal collagen deposition decreases in mice subjected to a second kidney insult. Animals were euthanized on day 21 post infection (p.i.), perfused, and the kidneys were collected for histologic analysis. Collagen deposition in the renal cortex was visualized using Picrosirius Red. Representative photomicrographs of (**A**) control_21d_, (**B**) control+BSA_21d_, (**C**) control+treated+BSA_21d_, (**D**) infected+treated_21d_, and (**E**) infected+treated+BSA_21d_ (*n* = 6 per group). Bar = 20 µm. Collagen deposition is quantified in (**F**). Values are expressed as a percentage of collagen deposition per area (means ± standard error). *Statistically significant compared with control_21d_. #Statistically significant compared with infected+treated _21d_ (*P*<0.05).

**Figure 9:**
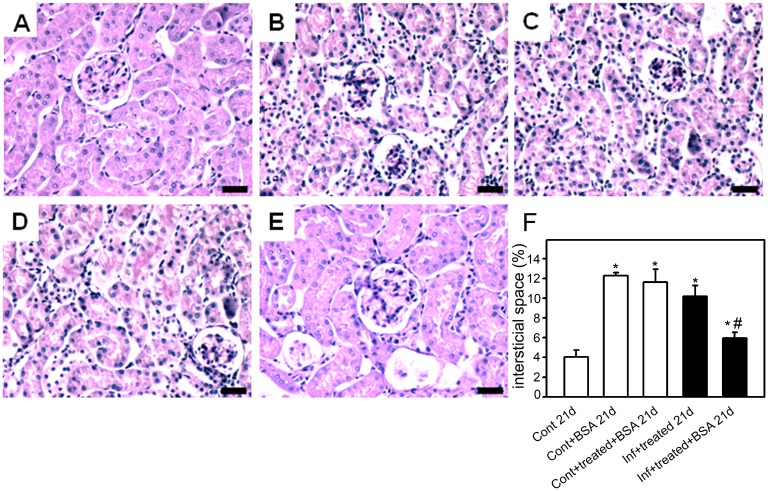
The size of the interstitial space is decreased in mice subjected to a second kidney insult. Animals were euthanized on day 21 post infection (p.i.), perfused, and the kidneys were collected for histologic analysis. The size if the interstitial space in the renal cortex was visualized using periodic acid−Schiff. Representative photomicrographs of (A) control_21d_, (B) control+BSA_21d_, (C) control+treated+ BSA_21d_, (D) infected+treated_21d_, and (E) infected+treated+BSA_21d_ (*n* = 6 per group). Bar = 20 µm. The size of the interstitial space is quantified in (**E**). Values are expressed as a percentage of interstitial space per area (means ± standard error) *Statistically significant compared with control_21d_. #Statistically significant compared with infected+treated _21d_ (*P*<0.05).

To confirm this hypothesis, the histomorphological parameters of the kidney, described above, were measured. The control+BSA_21d_ and infected+treated_21d_ groups showed a higher number of glomerular cells (Figure 7), increased collagen deposition (Figure 8), and more interstitial space (Figure 9). These changes were significantly lower in the infected+treated+BSA_21d_ group compared with the control+BSA_21d_ group. The treatment of the animals with antimalarial drugs did not change the histomorphological parameters on day 21 (Figures 3–5).

Our results show that treated mice infected with PbA are less susceptible to a second kidney insult compared with noninfected mice.

## Discussion

Malaria has been recognized as a parasitic disease that induces multiple organ dysfunction, including renal failure. The degree of kidney injury is directly associated with mortality in patients with malaria [5]. However, descriptions of the mechanisms involved in malaria-induced renal pathologies are rare in the literature. In the present work, we observed that treatment of PbA-infected mice with antimalarial drugs partially reverses kidney injury and completely abolishes parasitemia. PbA-infected mice treated with antimalarial drugs were less susceptible to a second kidney insult compared with noninfected animals that received the same treatment with antimalarials. These results open new perspectives on our understanding of the association of different pathologies in an endemic area.

Although there are important differences in terms of susceptibility to cerebral malaria in different mice strains, the biochemical parameters that reveal the degree of renal involvement are easily observed. Using PbA-infected BALB/c mice, Haines and Farmer [13] showed that the increase in parasitemia is associated with a reduction in renal GFR. Furthermore, Rui-Mei et al [14] showed that there is a direct correlation between parasitemia and proteinuria in PbA-infected C57BL/6 mice. In the present work, using PbA-infected C57BL/6 mice as a model of severe disease, we also observed a decrease in CCr, a marker of GFR, and a significant increase in proteinuria. These data show that the kidneys are a direct target during the pathogenesis of malaria and this pathology seems to be dissociated from cerebral damage, but is no less important. Supporting the crucial role of the kidneys in malaria, it has been observed that a high level of serum creatinine is among the majors factors associated with mortality in humans infected with *Plasmodium falciparum*, indicating a close correlation between both diseases [5]. Thus, treatment of the proteinuria per se could represent a coadjuvant therapy in the treatment of patients with malaria-induced AKI, increasing the probability of success in malaria treatment.

One important concern is the use of CCr as a marker of GFR, because it is secreted in the proximal tubule. However, CCr has been used as a good marker for GFR because it is an endogenous compound and invasive procedures can be avoided. CCr is not a good marker for GFR when the severe chronic kidney disease state occurs. Under these conditions, the level of creatinine secreted in the proximal tubule becomes as important as its filtration. We observed that the renal disease induced during malaria was reversed when the infection was eradicated by antimalarial treatment, indicating that the animals were not in a chronic renal disease state. Using C57BL/6 mice, the same mice used in the present work, Dunn et al [15] showed that the GFR measured by inulin clearance is very similar to that obtained by CCr. They also showed that the level of creatinine measured by high-performance liquid chromatography is similar to that measured by picric acid. Thus, it is plausible to postulate that the values of GFR measured by CCr in C57BL/6 mice are not significantly different from those obtained by other techniques and therefore do not lead to misinterpretation of our results.

During the progression of the malaria disease, the development of proteinuria is accompanied by an increase in pro-inflammatory cytokines and adhesion molecule ICAM-1 expression in peritubular capillaries because of the presence of parasite proteins in the membrane of infected red blood cells in the microvasculature [16]. On the other hand, it has been shown that overload of albumin in the proximal tubule promotes tubule interstitial injury leading to progression of renal disease through different pathways, including the accumulation of pro-inflammatory chemokines and cytokines such as TNF-α and IL-6 [12]. These results indicate that the protein overload in proximal tubules plays a role in tubule interstitial injury during renal disease induced by malaria and is more relevant than has been proposed. Therefore, the nature of the secondary kidney insult used in the present work is relevant because this model allows us to reproduce the protein overload in proximal tubule cells without any change in the GFR.

It is well established that malaria-induced renal injury is a consequence of parasite adhesion to renal endothelial cells as well as activation of the host immune response [3]. We observed that even when blood parasitemia was completely abolished by treatment with antimalarial drugs (infect+treated_21d_), renal injury was attenuated but still observed. The reason for this relies on exacerbated production of proinflammatory cytokines such as interferon-γ, tumor necrosis factor α, and interleukin-6, -1, and -8 produced during malaria infection. The same cytokines are involved in the pathogenesis of malaria-induced AKI and are correlated with the progression of proteinuria [14]. Infiltration of macrophages, lymphocytes, and malaria pigment deposition was observed in the kidney of PbA-infected C57BL/6 mice, a severe malaria model [17]. Here, we observed an increase in glomerular cell number, similar to Sinniah et al [17]. This result could be a consequence of cytokines and chemokines produced locally inducing glomerular infiltration during the course of infection.

In patients or an animal model with malaria, combined treatment with artesunate and mef loquine is sufficient to abolish parasitemia and attenuate cerebral malaria, contributing to improvement in survival [6]. Here, using similar treatment, total clearance of blood parasitemia was achieved and consequently improvement in animal survival. A recent study showed that treatment of PbA-infected albino mice with artesunate and chloroquine, two antimalarial drugs, attenuates interstitial infiltration of mononuclear cells and tubular nephrosis [7]. We showed that proteinuria and decreased GFR were partially but significantly reversed by treatment with antimalarial drugs in PbA-infected C57BL/6 mice. The antimalarial treatment also attenuates the increase in glomerular cell number, collagen deposition, and interstitial space. The effect of the antimalarial drugs was observed even 14 days after the end of the treatment (at day 21 p.i.) indicating that suspension of the treatment does not lead to recurrence of malaria-induced kidney injury.

One important question in relation to the treatment is the side effects of antimalarial drugs. Campos et al [18] observed that treatment of healthy Wistar rats with a 12 mg/kg artesunate promotes a decrease in the GFR. On the other hand, we observed that treatment of noninfected mice with antimalarial drugs did not change the histomorphological parameters measured 14 days after treatment. Moreover, the increased BUN/serum creatinine ratio observed in this group of mice after treatment could be an indication of liver injury more than a renal effect.

An interesting result obtained in the present work is the observation that infected mice treated with antimalarial drugs are less susceptible to a second kidney insult. Antimalarial drugs have been used in the treatment of other pathologies besides malaria. The administration of artemisinin protects against sepsis in mice challenged with lipopolysaccharide by decreasing the secretion of proinflammatory cytokines [19]. However, a possible renal protection effect of antimalarials during a second kidney insult can be rule out because there is no difference in renal injury observed in the noninfected (control+treated+BSA_21d_) and kidney insult (control+BSA_21d_) groups.

Malaria and septic shock have important similarities in relation to induction of AKI [3], [14], [20]. Contrary to what was observed with sepsis, we showed a reversal of important parameters such as GFR, serum creatinine, collagen deposition, and size of interstitial space, indicating that malaria-surviving mice are less susceptible to a second kidney injury. These observations indicate that in malaria, an AKI-induced response occurs by a specific unknown mechanism that is different from sepsis.

The pathology of severe malaria has been extensively explored, however mortality induced by *Plasmodium falciparum* remains high, despite the advances in therapy and prevention of disease [21]. All the pathologic changes that result from malaria could induce signaling pathways that definitely represent a fruitful area for further studies. An in-depth view of those mechanisms could open new perspectives on therapeutic interventions, especially in the case of repeat *P. falciparum* infection or the combination of different pathologies in endemic areas.

## Supporting Information

Figure S1 **Effect of antimalarial treatment on urinary protein.** Animals were euthanized on day 5 or day 21 post infection and urine samples were resolved on sodium dodecyl sulfate polyacrylamide gel electrophoresis. Protein analysis was based on the intensity of Coomassie blue staining. Urinary samples were collected from the different experimental groups as depicted in the figure.
(TIF)
